# 血液重症监护病房中序贯器官衰竭评估及其动态变化预测死亡率的价值

**DOI:** 10.3760/cma.j.cn121090-20241130-00510

**Published:** 2025-01

**Authors:** 佳婧 王, 建 张, 斌 张, 云聪 曹, 怡麟 郭, 沛然 于, 晓庆 张, 晓娟 张, 毅军 宋

**Affiliations:** 1 中国医学科学院血液病医院（中国医学科学院血液学研究所），血液与健康全国重点实验室，国家血液系统疾病临床医学研究中心，细胞生态海河实验室，天津 300020 State Key Laboratory of Blood and Health, National Clinical Research Center for Blood Diseases, Haihe Laboratory of Cell Ecosystem, Institute of Hematology & Blood Diseases Hospital, Chinese Academy of Medical Sciences & Peking Union Medical College, Tianjin 300020, China; 2 天津医学健康研究院，天津 301600 Tianjin Institutes of Health Science, Tianjin 301600, China; 3 天津医科大学，天津 300070 Tianjin Medical University, Tianjin 300070, China; 4 天津中医药大学，天津 301617 Tianjin University of Traditional Chinese Medicine, Tianjin 301617, China

**Keywords:** 血液病, 危重病, 重症监护病房, 死亡率, 序贯器官衰竭评估, Hematologic diseases, Critical illness, Intensive care units, Mortality, Sequential organ failure assessment

## Abstract

**目的:**

探讨序贯器官衰竭评估（SOFA）评分及其动态变化（ΔSOFA）在血液重症监护病房（HCU）中预测死亡率的价值。

**方法:**

回顾性收集2024年5月至2024年6月中国医学科学院血液病医院重症医学诊疗中心收治的79例血液病危重症患者的临床数据，计算患者自住院于HCU前2 d至后3 d的SOFA评分及ΔSOFA。应用受试者工作特征曲线（ROC）研究SOFA评分及ΔSOFA在死亡率预测中的价值。

**结果:**

79例患者的HCU死亡率为54.4％。死亡组全体患者、白血病患者和造血干细胞移植（HSCT）患者住院第1～3天SOFA（D_1、D_2、D_3）评分和第1天ΔSOFA（ΔD_1）均较非死亡组明显增高（*P*值均<0.05）。ROC曲线分析显示，D_1、D_2、D_3评分和ΔD_1均可预测患者死亡率（*P*<0.001），曲线下面积（AUC）分别为0.786、0.866、0.901、0.843，灵敏度分别为74.36％、57.89％、62.85％、86.84％，特异度分别为70.00％、100％、100％、67.65％。HSCT组中，D_-1、D_1、D_2、D_3评分和ΔD_1均可预测HCU死亡率，AUC分别为0.833、0.794、0.871、0.846、0.795；灵敏度分别为100％、85.71％、71.43％、57.14％、57.14％；特异度分别为73.33％、70.59％、91.33％、100％、100％。白血病组中，D_1、D_2、D_3评分和ΔD_1可预测HCU死亡率，AUC分别为0.760、0.829、0.846、0.756；灵敏度分别为71.43％、78.57％、53.85％、71.43％；特异度分别为76.19％、78.95％、100％、63.16％。对于全体患者，D_3评分特异度最高，ΔD_1灵敏度最高。对于HSCT组和白血病组患者，D_1和D_3评分的灵敏度及特异度均高于ΔD_1。

**结论:**

对于住院于HCU的血液危重症、白血病和HSCT患者，D_1、D_2、D_3评分和ΔD_1与HCU死亡率明显相关。

## Abstract (en)

**Objective:**

To investigate the value of Sequential Organ Failure（SOFA）score and its dynamics（ΔSOFA）in predicting mortality in hematology care unit（HCU）.

**Methods:**

A retrospective clinical study was conducted on 79 critically ill hematologic patients admitted to the Center for Critical Care Medicine, Institute of Hematology & Blood Diseases Hospital, Chinese Academy of Medical Sciences, between May and June 2024. SOFA scores and ΔSOFA were calculated from 2 days before HCU admission to 3 days after HCU admission. The predictive value of SOFA and ΔSOFA in mortality was assessed using receiver operating characteristic（ROC）curve analysis.

**Results:**

Among the 79 patients, the HCU mortality rate was 54.4％. The SOFA scores on days 1–3（D1, D2, and D3）and ΔSOFA on day 1（ΔD_1）of all patients, leukemia patients and hematopoietic stem cell transplantation（HSCT）patients were significantly higher in the death group compared with the non-death group（all *P*<0.05）. ROC curve analysis revealed that the D_1, D_2, D_3 scores, and ΔD_1 significantly predicted mortality（*P*<0.001）, with areas under the curve（AUCs）of 0.786, 0.866, 0.901, and 0.843, respectively. The sensitivity values were 74.36％, 57.89％, 62.85％, and 86.84％, while specificity values were 70％, 100％, 100％, and 67.65％, respectively. In the HSCT group, the D_-1, D_1, D_2, D_ 3, scores and ΔD_1 were predictive of HCU mortality, with AUCs of 0.833, 0.794, 0.871, 0.846, and 0.795, respectively. Sensitivity values for these scores were 100％, 85.71％, 71.43％, 57.14％, and 57.14％, while specificity values were 73.33％, 70.59％, 91.33％, 100％, and 100％, respectively. In the leukemia group, the D_1, D_2, D_3 scores, and ΔD_1 were predictive of HCU mortality, with AUCs of 0.760, 0.829, 0.846, and 0.756, respectively. Sensitivity values were 71.43％, 78.57％, 53.85％, and 71.43％, while specificity values were 76.19％, 78.95％, 100％, and 63.16％, respectively. For all patients, the D_3 score exhibited the highest specificity, while the ΔD_1 demonstrated the highest sensitivity. For patients in both the HSCT and leukemia groups, the sensitivity and specificity values of the D_1 and D_3 scores exceeded those of the ΔD_1.

**Conclusion:**

For patients with hematologic critical illness, including leukemia and those undergoing HSCT hospitalized in the HCU, D_1, D_2, D_ 3 scores and ΔD_1 are significantly associated with HCU mortality.

新诊断血液病患者1年内出现危重症的风险约15％，需要在重症监护病房（ICU）内完成治疗。急性白血病、淋巴瘤和造血干细胞移植（HSCT）后患者危重症出现的比例更高，分别为22％、17％和25％[1]–[5]。血液病危重症患者的预后差，住院死亡率高达80％，ICU死亡率也高达40％～50％[6]–[7]。近十年来，随着血液病重症救治水平提高，恶性血液病ICU的住院率逐年提高[1],[8]，其生存率增加到51％（46％～66％）[9]–[10]。因此，对于恶性血液病患者，血液重症监护病房（HCU）救治已成为必不可少的临床需求。寻找到能预测HCU死亡率的临床指标，有利于早期积极干预和提高生存率，是血液重症当前研究的热点之一。

作为评价脓毒血症危重症严重程度和预后的筛查工具，序贯器官衰竭评估评分（SOFA评分）及其动态变化（ΔSOFA）已开始在综合ICU中用于预测恶性血液病的预后[11]–[13]。Platon等[14]对318例HSCT患者的预后影响因素开展了单中心回顾性研究，73例HSCT患者需住院于ICU，ICU死亡率为40.3％，ICU住院第1～3天SOFA评分增加在HSCT的死亡预测中有价值（*HR*＝1.52，95％ *CI* 1.08～2.15，*P*＝0.01）。Geerse等[15]回顾性研究了综合ICU中86例恶性血液病患者的预后相关因素，与幸存组相比，死亡组住院第1天SOFA评分显著升高（11.5±3.1对8.5±3.0）；ΔSOFA（住院第7天SOFA评分−住院当天SOFA评分）≥2分组的死亡率显著高于ΔSOFA＝0分组和ΔSOFA<0分组（72％对58％对21％）。SOFA评分及ΔSOFA可能作为评价综合ICU中恶性血液病患者预后的指标之一。但综合ICU中血液危重症的诊疗仍有待进一步规范，血液病单因素导致的ICU死亡率和预后的特异性评价体系尚未建立。

HCU在保障血液病的规范性和续贯治疗下，能及时、高效地完成危重症诊疗，降低其死亡率[16]。在HCU中明确死亡率的预测指标有重要的临床和社会价值。本研究回顾性收集中国医学科学院血液病医院血液危重症患者住院于HCU前后的SOFA评分和ΔSOFA，研究其在HCU死亡率预测中的价值。

## 病例与方法

1. 病例：回顾性纳入2024年5月至2024年6月在中国医学科学院血液病医院重症医学诊疗中心住院治疗的血液病危重症患者。纳入标准：年龄≥18岁；血液病首诊患者；首次入住HCU。排除标准：非首次入住HCU；非HCU住院史；经急诊直接入住HCU；在HCU内住院时长<72 h；因各种原因在HCU治疗中断的患者。本研究经中国医学科学院血液病医院医学伦理委员会批准（伦理批号：QTJC2024062-EC-1）。

收集患者的性别、年龄、血液病类型、住院期间支持治疗（机械通气、血管收缩药和肾脏替代治疗等）、HCU住院时间、HCU死亡率等临床资料。以HCU死亡作为临床结局的判断指标，将纳入的血液危重症患者分为死亡组和非死亡组。

2. 调查工具与方法：SOFA评分包括6个系统（呼吸系统、心血管系统、凝血系统、神经系统、肝脏系统和肾脏系统）的指标评分，每项评分为0～4分，总计24分[17]。ΔSOFA为评估当日SOFA评分与前1日SOFA评分的差值。计算所有患者住院于HCU前2 d至后3 d的SOFA（共5 d，分别表示为D_-2、D_-1、D_1、D_2、D_3）评分和ΔSOFA（共4 d，分别表示为ΔD_-1、ΔD_0、ΔD_1、ΔD_2）。分别比较死亡组和非死亡组的SOFA评分和ΔSOFA差异。选择HCU中最常见的类型：白血病和HSCT作为亚组，在组内进一步分析SOFA评分和ΔSOFA对于HCU死亡率的预测价值。

3. 随访：以检索患者住院电子病历系统方式获取随访数据，中位随访时间为12（3～45）d，随访截止时间为2024年8月15日。

4. 统计学处理：采用SPSS 20.0分析数据。计数资料数据用例数（百分比）描述，组间比较采用*χ*^2^检验。符合正态分布的计量资料用均数±标准差描述，符合偏态分布的计量资料用*M*（范围）描述，组间比较采用独立样本*t*检验，组内比较采用重复因素方差分析。应用ROC曲线评价住院于HCU前2 d至后2 d的SOFA评分及ΔSOFA对HCU死亡率的预测价值，计算曲线下面积AUC。*P*<0.05为差异有统计学意义。

## 结果

1. 临床特征：自2024年5月至2024年6月，94例成人血液病危重症患者由普通病房转入HCU治疗。因于HCU住院时长不满72 h排除4例，因于HCU住院期间自动出院排除11例，最终纳入79例患者，总体死亡率为54.4％；白血病患者35例（44.3％），HCU死亡率为60.0％；HSCT患者24例（34.8％），HCU死亡率为70.8％。79例患者的临床特征见表1，其SOFA评分和ΔSOFA见表2。

**表1:** 79例血液病危重症患者的临床特征

特征	总体（79例）	死亡组（43例）
年龄［岁，*M*（范围）］	51（18～87）	52（18～87）
性别（例，男/女）	38/41	22/21
血液病类型［例（％）］		
白血病	35（44.3）	21（48.8）
淋巴瘤	11（13.9）	7（16.3）
多发性骨髓瘤	6（7.6）	3（7.0）
骨髓增生异常综合征	9（11.4）	4（9.3）
再生障碍性贫血	7（8.9）	3（7.0）
血小板减少性紫癜	4（5.1）	0（0）
其他	7（8.9）	5（11.6）
造血干细胞移植［例（％）］		
是	24（30.4）	17（39.5）
否	55（69.6）	26（60.5）
HCU住院病因［例（％）］		
脓毒血症	41（51.9）	25（58.1）
休克	14（17.7）	8（18.6）
急性呼吸衰竭	18（22.8）	16（37.2）
急性心功能衰竭	20（25.3）	13（30.2）
急性肝功能衰竭	14（17.7）	10（23.3）
急性肾功能衰竭	18（22.8）	8（18.6）
意识障碍	23（29.1）	12（27.9）
癫痫持续状态	10（12.7）	4（9.3）
HCU内生命支持治疗［例（％）］		
机械通气	34（43.0）	32（74.4）
气管插管	28（35.4）	28（65.1）
非侵入性机械通气	23（29.1）	21（48.8）
血管收缩药	32（40.5）	30（69.8）
连续性肾脏替代治疗	29（36.7）	17（39.5）
HCU治疗时间［d，*M*（范围）］	9（3～45）	5（3～45）

**注** HCU：血液重症监护病房

**表2:** 血液重症监护病房死亡组与非死亡组患者SOFA评分及其动态变化（ΔSOFA）比较（分，*x*±*s*）

类型	量表	时间	全体	死亡组	非死亡组	*t*值^a^	*P*值^a^
全体	SOFA评分	D_-2	7.28±0.03	7.44±0.39	7.00±0.56	0.67	0.510
		D_-1	8.12±0.03	8.26±0.42	7.92±0.66	0.45	0.650
		D_1	10.61±0.05	12.90±0.55	8.44±0.65	5.23	<0.001
		D_2	10.33±0.06	13.55±0.64	7.58±0.61	6.73	<0.001
		D_3	10.07±0.06	13.60±0.61	7.54±0.62	6.78	<0.001
	ΔSOFA	ΔD_-1	0.90±0.03	0.91±0.32	0.87±0.48	0.08	0.936
		ΔD_0	3.58±0.04	4.28±0.64	2.54±0.49	2.27	0.027
		ΔD_1	0.01±0.02	1.12±0.29	−0.97±0.23	5.74	<0.001
		ΔD_2	0.37±0.01	0.64±0.19	0.17±0.17	1.84	0.070
HSCT	SOFA评分	D_-2	7.52±0.23	8.07±0.51	7.00±0.63	1.11	0.283
		D_-1	8.03±0.01	9.20±0.71	6.60±0.68	1.98	0.062
		D_1	11.61±0.07	13.06±1.03	8.14±1.57	2.59	0.017
		D_2	12.32±0.36	14.07±1.18	7.43±1.63	3.23	0.004
		D_3	11.87±0.06	13.62±0.95	8.14±1.77	3.00	0.008
	ΔSOFA	ΔD_-1	0.92±0.13	1.13±0.59	−0.40±0.24	1.46	0.163
		ΔD_0	3.58±0.04	3.53±1.05	3.40±1.21	0.07	0.947
		ΔD_1	0.08±0.05	1.40±0.56	−0.71±0.64	2.27	0.035
		ΔD_2	0.37±0.01	0.54±0.33	0.71±0.29	0.35	0.730
白血病	SOFA评分	D_-2	7.02±0.42	7.12±0.61	6.50±0.87	0.597	0.556
		D_-1	8.17±0.34	8.29±0.70	8.10±1.40	0.14	0.891
		D_1	11.72±0.45	12.84±0.94	9.14±1.11	2.55	0.016
		D_2	12.32±0.36	13.40±1.00	8.57±0.98	3.44	0.002
		D_3	11.94±0.07	14.62±0.87	8.23±0.94	4.97	<0.001
	ΔSOFA	ΔD_-1	1.09±0.03	0.67±0.32	1.60±1.02	1.07	0.293
		ΔD_0	3.56±0.05	4.31±0.78	2.00±0.92	1.83	0.079
		ΔD_1	0.87±0.12	1.11±0.45	−0.57±0.43	2.61	0.014
		ΔD_2	0.35±0.21	0.75±0.18	0.23±0.32	1.37	0.183

**注** SOFA：序贯器官衰竭评估；^a^死亡组与非死亡组比较；D_-2：住院前2 d；D_-1：住院前1 d；D_1：住院第1 d；D_2：住院第2 d；D_3：住院第3 d；ΔD_-1：住院前1 d；ΔD_0：住院当天；ΔD_1：住院后1 d；ΔD_2：住院后2 d；HSCT：造血干细胞移植

2. 死亡组与非死亡组SOFA评分、ΔSOFA比较：全体患者、HSCT患者和白血病患者中，死亡组与非死亡组住院于HCU前2 d至后3 d SOFA评分的动态变化及组间差异比较见图1、表2。

**图1:**
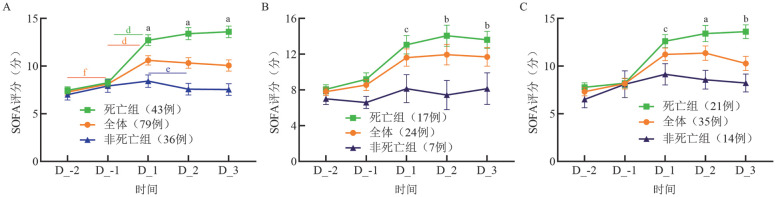
全体、死亡组与非死亡组患者住院于血液重症监护病房的SOFA评分动态变化 A 全体患者；B 造血干细胞移植患者；C 白血病患者 **注** SOFA：序贯器官衰竭评估；死亡组与非死亡组SOFA评分比较，^a^*P*<0.001，^b^*P*<0.01，^c^*P*<0.05；同组患者不同日期SOFA评分比较，^d^*P*<0.001，^e^*P*<0.01，^f^*P*<0.05；D_-2：住院前2 d；D_-1：住院前1 d；D_1：住院第1 d；D_2：住院第2 d；D_3：住院第3 d

死亡组的D_1、D_2和D_3评分及ΔD_0、ΔD_1均较非死亡组显著增高（*P*值均<0.05）。在死亡组中，D_1较D_-1评分显著增高（*P*<0.001），而在非死亡组中，D_2较D_1评分显著降低（*P*＝0.004）。在HSCT和白血病亚组中，死亡组住院D_1、D_2、D_3评分和ΔD_1均显著高于非死亡组（表2）。

3. SOFA评分预测死亡率的ROC曲线：ROC曲线分析显示，D_1、D_2、D_3评分和ΔD_1均可预测患者死亡率（*P*<0.001），曲线下面积（AUC）分别为0.786、0.866、0.901、0.843，灵敏度分别为74.36％、57.89％、62.85％、86.84％，特异度分别为70.00％、100％、100％、67.65％（图2、表3）。

**图2:**
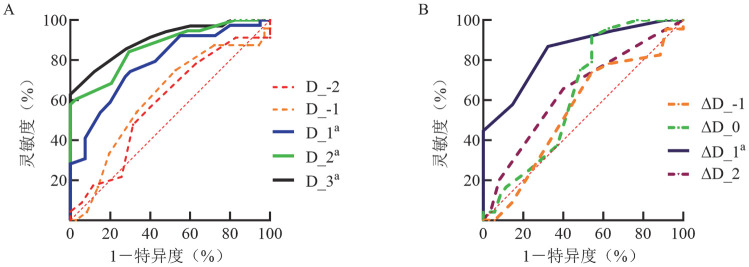
SOFA评分（A）及其动态变化（ΔSOFA）（B）预测79例血液危重症患者死亡率的ROC曲线 **注** SOFA：序贯器官衰竭评估；实线表示差异有统计学意义，虚线表示差异无统计学意义；^a^*P*<0.001；D_-2：住院前2 d；D_-1：住院前1 d；D_1：住院第1 d；D_2：住院第2 d；D_3：住院第3 d；ΔD_-1：住院前1 d；ΔD_0：住院当天；ΔD_1：住院后1 d；ΔD_2：住院后2 d

**表3:** SOFA评分及其动态变化（ΔSOFA）预测血液病危重症患者死亡率的诊断效能

类型	量表	截断值（分）	AUC	95％ *CI*	灵敏度（％）	特异度（％）	约登指数（％）	*P*值
全体	D_-2	7	0.584	0.433～0.735	56.52	60.00	16.52	0.283
	D_-1	8	0.611	0.483～0.759	75.00	47.22	22.22	0.148
	D_1	11	0.786	0.686～0.886	74.36	70.00	44.36	<0.001
	D_2	7	0.866	0.785～0.947	57.89	100	57.89	<0.001
	D_3	8	0.901	0.826～0.975	62.86	100	62.86	<0.001
	ΔD_-1	0	0.548	0.396～0.701	73.91	45.71	19.62	0.535
	ΔD_0	5	0.635	0.494～0.776	91.67	45.71	37.38	0.081
	ΔD_1	1	0.843	0.754～0.932	86.84	67.65	54.49	<0.001
	ΔD_2	0	0.642	0.500～0.785	65.71	60.00	25.71	0.062
HSCT	D_-2	8	0.713	0.492～0.935	100	46.67	46.67	0.163
	D_-1	8	0.833	0.657～1.000	100	73.33	73.33	0.029
	D_1	11	0.794	0.587～1.000	85.71	70.59	56.30	0.026
	D_2	8	0.871	0.704～1.000	71.43	91.33	64.76	0.006
	D_3	8	0.846	0.656～1.000	57.14	100	57.14	0.013
	ΔD_-1	0	0.727	0.507～0.947	100	46.67	46.67	0.138
	ΔD_0	3	0.513	0.239～0.788	60.00	53.33	13.33	0.930
	ΔD_1	−1	0.795	0.572～1.000	57.14	100	57.14	0.029
	ΔD_2	0	0.522	0.264～0.780	100	15.38	15.38	0.874
白血病	D_-2	6	0.699	0.499～0.899	69.23	77.78	47.01	0.063
	D_-1	7	0.680	0.471～0.889	76.92	68.42	45.34	0.088
	D_1	10	0.760	0.584～0.937	71.43	76.19	47.62	0.010
	D_2	10	0.829	0.678～0.980	78.57	78.95	57.52	0.001
	D_3	8	0.846	0.686～1.000	53.85	100	53.85	0.003
	ΔD_-1	0	0.534	0.318～0.750	69.23	44.44	13.67	0.749
	ΔD_0	6	0.640	0.448～0.832	100	36.84	36.84	0.186
	ΔD_1	0	0.756	0.584～0.924	71.43	63.16	34.59	0.013
	ΔD_2	0	0.644	0.421～0.867	61.54	66.67	28.21	0.221

**注** SOFA：序贯器官衰竭评估；D_-2：住院前2 d的SOFA评分；D_-1：住院前1 d的SOFA评分；D_1：住院第1 d的SOFA评分；D_2：住院第2 d的SOFA评分；D_3：住院第3 d的SOFA；ΔD_-1：住院前1 d的ΔSOFA；ΔD_0：住院当天ΔSOFA；ΔD_1：住院后1 d的ΔSOFA；ΔD_2：住院后2 d的ΔSOFA；HSCT：造血干细胞移植

在HSCT组中，D_-1、D_1、D_2、D_3评分和ΔD_1均可预测HCU死亡率，AUC分别为0.833、0.794、0.871、0.846、0.795；灵敏度分别为100％、85.71％、71.43％、57.14％、57.14％；特异度分别为73.33％、70.59％、91.33％、100％、100％（图3A、B）（表3）。

**图3:**
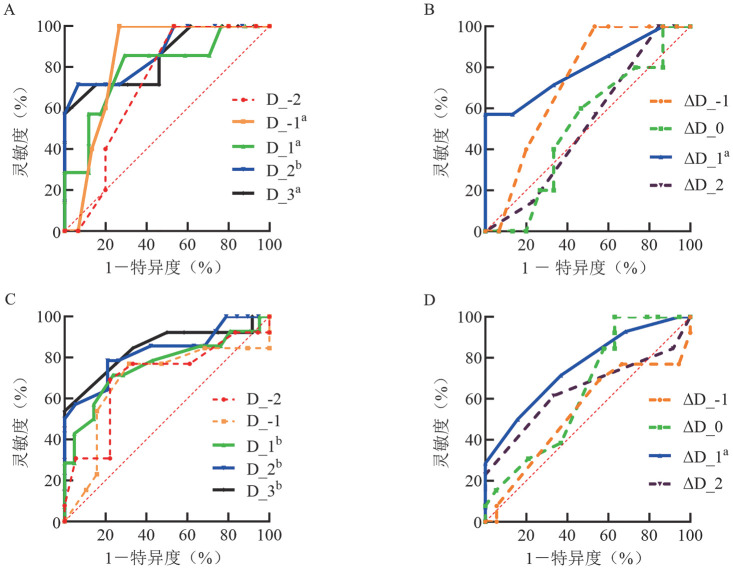
SOFA评分及其动态变化（ΔSOFA）预测HSCT和白血病患者血液重症监护病房死亡率的ROC曲线 A SOFA评分预测HSCT患者；B ΔSOFA预测HSCT患者；C SOFA评分预测白血病患者；D ΔSOFA预测白血病患者 **注** SOFA：序贯器官衰竭评估；实线表示差异有统计学意义，虚线表示差异无统计学意义；HSCT：造血干细胞移植；^a^*P*<0.05，^b^*P*<0.01；D_-2：住院前2 d；D_-1：住院前1 d；D_1：住院第1 d；D_2：住院第2 d；D_3：住院第3 d；ΔD_-1：住院前1 d；ΔD_0：住院当天；ΔD_1：住院后1 d；ΔD_2：住院后2 d

在白血病组中，D_1、D_2、D_3评分和ΔD_1可预测HCU死亡率，AUC分别为0.760、0.829、0.846、0.756；灵敏度分别为71.43％、78.57％、53.85％、71.43％；特异度分别为76.19％、78.95％、100％、63.16％（图3C、D）（表3）。

## 讨论

本研究显示，HCU死亡率为54.4％，其中，HSCT组为70.8％，白血病组为60.0％。D_1、D_2、D_3评分和ΔD_1在血液危重症死亡率预测中有重要价值。

急性白血病、淋巴瘤和HSCT等血液危重症患者常需在ICU接受抢救治疗[18]–[19]，加拿大一项多中心前瞻性研究显示，在414例恶性血液病ICU住院患者中，急性白血病患者占51％、淋巴瘤或多发性骨髓瘤患者占38％、HSCT患者占40％[3]。白细胞增多和淤积、肿瘤溶解综合征、弥散性血管内凝血、肿瘤诱导的微血管病性溶血性贫血、继发性噬血细胞淋巴组织细胞增生症、血浆高黏滞综合征、骨髓瘤相关并发症和急性移植物抗宿主病等并发症累及呼吸、循环、肾脏及神经等系统，造成急性呼吸衰竭、脓毒血症、急性肾功能衰竭等严重并发症是患者住院于ICU的主要原因[4],[19]–[20]。

综合ICU中常应用SOFA评分和ΔSOFA预测患者的不良结局[11]–[13]。Ferreira等[21]发现，住院期间SOFA评分平均值和最高值是预测综合ICU内脓毒血症患者死亡率的有效指标，住院于ICU后48 h内ΔSOFA增加值≥30％，脓毒症病死率≥50％。Raith等[13]的研究纳入184 875例首次住院于综合ICU的疑似感染患者，研究住院后24 h内ΔSOFA、全身炎症反应综合征（SIRS）评分和快速SOFA（qSOFA）评分在住院死亡率预测中的价值，发现ΔSOFA≥2分（AUC＝0.753，95％ *CI* 0.750～0.757）在死亡率预测中的特异度最高。Vogel等[22]的回顾性研究纳入52例伴肝硬化的重症急性胰腺炎（LC-AP）患者，研究SOFA评分对LC-AP组患者结局的预测价值，发现住院当日SOFA评分可预测LC-AP患者的不良结局（AUC＝0.86±0.07，*P*<0.05）。由此可见，在综合ICU脓毒血症和非感染性疾病中，SOFA评分和ΔSOFA均有助于预测疾病的危重程度和预后，分值越高，死亡风险越大。

评价HCU患者预后的研究目前尚不充分。MacPhail等[8]的研究纳入因脓毒血症住院于综合ICU的患者，研究恶性血液病组（17 313例）和非血液系统恶性肿瘤组（265 314例）的死亡风险差异，发现恶性血液病组住院死亡率显著高于非血液系统恶性肿瘤组（31.2％对17.0％，*P*<0.001）；D_1分值与恶性血液病组死亡率显著相关（*P*<0.001）。Cornet等[23]的研究显示，在ICU住院患者中，血液病组（58例）的死亡率为62.1％，36例死亡患者的SOFA评分（11.2±3.5，住院第1～4天SOFA评分平均值）对其死亡率有预测价值（AUC＝0.84，95％ *CI* 0.74～0.94，*P*<0.001）。Lamia等[24]收集了住院于ICU的92例恶性血液病患者临床数据，回顾性研究住院前2 d至第1天SOFA评分与死亡率的关系，发现住院第1天SOFA可有效预测死亡率（*OR*＝1.35，95％ *CI* 1.17～1.56，*P*<0.001）。与既往研究相比，本研究纳入患者的主要病因为恶性血液病，均为血液科普通病房转诊至HCU，治疗方案均根据血液病指南规范，因此研究人群更为合理。结果显示，住院早期SOFA评分和ΔSOFA对血液危重症患者的死亡率有预测价值，D_3评分>8分特异度最高，ΔD_1>1分灵敏度最高，有必要进一步探讨其可能机制。

在HSCT和白血病患者出现危重症的风险高于其他血液病，但SOFA评分预测其死亡率价值的研究较少。Díaz-Lagares等[25]回顾性研究了82例HSCT患者住院于ICU第1、3、5天的SOFA评分与其预后的关系，与非死亡组（22例）相比，死亡组（60例）的D_1评分（中位数为9，*HR*＝1.11，95％ *CI* 1.04～1.02，*P*＝0.002）和ΔSOFA（第5天减第1天）≥0分（*HR*＝2.13，95％ *CI* 1.03～4.39，*P*＝0.04）与院内死亡率相关。Neumann等[26]的研究纳入在综合ICU住院的64例HSCT患者，与非死亡组相比，死亡组患者住院第1天的SOFA评分可预测其死亡率（*OR*＝10.1，95％ *CI* 2.64％～38.70％，*P*<0.001）。Platon等[14]的回顾性研究显示，住院于ICU的73例HSCT患者的死亡率为44％，ΔSOFA（住院第3天减第1天）≥0分在死亡率预测中有重要价值（*HR*＝1.52，95％ *CI* 1.08～2.15，*P*＝0.01），住院第4～7天SOFA评分对死亡率无预测价值（*P*>0.05）。Heger等[27]的研究纳入住院于ICU的69例AML患者，发现第1天SOFA评分≥7分可作为ICU死亡率预测的最佳截断值（*P*<0.001）。与国内外研究结果类似，本研究发现HSCT组患者中D_1>11分、D_2>8分、D_3>8分和ΔD_1>-1分，白血病组患者中D_1>10分、D_2>10分、D_3>8分和ΔD_1>0分对死亡率预测具有重要价值。本研究还收集了住院于ICU前2 d的评分数据，结果提示，HSCT患者D_-1评分也可有效预测死亡率，且灵敏度最高，最佳截断值为8分。D_-1评分预测死亡率结果阳性，对于识别HSCT患者疾病严重程度、积极干预和改善预后具有重要价值。

与ICU中非血液系统疾病相比，血液危重症的专业诊疗要求更强、ICU住院病因更复杂、疾病危重程度及死亡率均更高[28]–[30]，急需可靠的评分系统对血液危重症死亡率进行早期预测，更科学地指导和安排临床诊疗。本研究全程收集血液危重症患者住院于HCU前2 d至后3 d的SOFA评分和ΔSOFA，发现D_1、D_2、D_3评分和ΔD_1与其HCU死亡率明显相关，对HCU死亡率有预测价值。此外，对于HSCT组患者，D_-1评分对于HCU死亡率有预测价值。上述结果均提示在未来有必要开展多中心、大样本、前瞻性临床研究进一步验证。
